# The Benefits of Technology for Engaging Aging Adults: Findings From the PRISM 2.0 Trial

**DOI:** 10.1093/geroni/igae042

**Published:** 2024-04-25

**Authors:** Sara J Czaja, Neil Charness, Wendy A Rogers, Joseph Sharit, Jerad H Moxley, Walter R Boot

**Affiliations:** Center on Aging and Behavioral Medicine, Weill Cornell Medicine, New York, New York, USA; Department of Psychology, Florida State University, Tallahassee, Florida, USA; College of Applied Health Sciences, University of Illinois at Urbana-Champaign, Urbana, Illinois, USA; College of Engineering, University of Miami, Coral Gables, Florida, USA; Center on Aging and Behavioral Medicine, Weill Cornell Medicine, New York, New York, USA; Center on Aging and Behavioral Medicine, Weill Cornell Medicine, New York, New York, USA

**Keywords:** Social engagement, Loneliness, Technology access, Living environment

## Abstract

**Background and Objectives:**

Technology has potential for providing support for aging adults. This study evaluated the Personal Reminder Information and Social Management 2.0 (PRISM 2.0) software, in terms of enhancing social engagement and quality of life, and decreasing loneliness among older adults.

**Research Design and Methods:**

The randomized field trial conducted in diverse living contexts (rural locations, senior housing, and assisted living communities [ALC]). Two hundred and forty-five adults, aged 64 to 99 years, were randomly assigned to the PRISM 2.0 (integrated software system designed for aging through an iterative design process) or a Standard Tablet (without PRISM) Control condition, where participants received the same amount of contact and training as those in the PRISM 2.0 condition. Primary outcomes included measures of loneliness, social support, social connectedness, and quality of life. Secondary outcomes included measures of social isolation, mobile device proficiency, and technology readiness. Data were collected at baseline and 6 and 9 months postrandomization. This article focuses on the 6-month outcomes due to coronavirus disease 2019-related data challenges at 9 months.

**Results:**

Contrary to our hypothesis, participants in rural locations and senior housing in both conditions reported less loneliness and social isolation, and greater social support and quality of life at 6 months, and an increase in mobile device proficiency. Participants in the ALCs in both conditions also evidenced an increase in mobile device proficiency. Improvements in quality of life and health-related quality of life were associated with decreases in loneliness.

**Discussion and Implications:**

This study provides compelling evidence about the benefits of technology for older adults in terms of enhancing social outcomes and quality of life. However, the findings also underscore that for technology applications to be successful, they need to be adapted to the abilities and needs of the user group and instructional support needs to be provided.

**Clinical Trials Registration #:**

NCT03116399

**Translational Significance:** Older adults often experience challenges with lack of social connectivity and loneliness. This field trial demonstrated that technology applications may help reduce social isolation, loneliness, and enhance quality of life. Further, exposure to and training on the use of technology can enhance technology skills, which can reduce the digital divide and associated consequences even among older adults with health and cognitive challenges. Overall, our findings demonstrate that technology applications are scalable and can be beneficial to diverse populations of aging adults living in diverse contexts. The findings also provide insight into implementation challenges associated with deploying technology in different contexts.

Although an age-related digital divide remains, especially among some populations of older adults such as those with disabilities or in rural locations, technology use has generally increased among persons aged 65 and older. According to recent data from the Pew Research Center (Faverio, 2022), currently 75% of those aged 65 and older use the internet as compared to 63% in 2015, and 64% indicate that they have home broadband as compared to 40% in 2015.

Much has been written about the potential benefits of technology for aging adults on health and outcomes such as social engagement. This is especially true given the recent coronavirus disease 2019 (COVID-19) pandemic, when technology was essential to accessing healthcare and services and remaining socially connected. For example, data from a survey study (McClain et al., 2012) indicated that the share of older adults (aged 65+) who felt that access to the internet was essential increased during the pandemic from 31% in 2020 to 38% in 2021, and an additional 48% indicated that internet access was important. However, the findings also indicated that older adults had lower “tech readiness” (willingness to adopt and use emerging technologies; Parasuraman, 2000) and lower confidence regarding use of technology applications such as the internet. Haase and colleagues (2021) examined the uptake and acceptance of technology for socialization during the pandemic among a sample of 400 older adults in British Columbia. Fifty-six percent of the sample reported that they used technology differently to connect with others, and ~60% reported that they adopted new technology. Facilitators to use included knowledge of technologies, reliance on others (e.g., family), technology accessibility (e.g., usability, clear instructions), and social motivation. Key barriers to use included lack of access and interest, and health barriers such as arthritis or cognitive impairments. Findings regarding barriers to technology uptake among aging adults confirm those of others (e.g., Charness & Boot, 2022), indicating that income, infrastructure, and attitudinal issues also play a role in the age-related digital divide.

Numerous studies have examined the value of technology for older adults and issues associated with barriers to access. Overall, the results of these studies are encouraging. For example, several studies have found that use of technology, such as the internet, among older adults results in reductions in depressive symptoms (e.g., Chopik, 2016; Cotten et al., 2012, 2014; Wang et al., 2019) and higher life satisfaction (e.g., Lifshitz et al., 2018; Seifert et al., 2017).

Use of technology by older adults can result in improvements in social outcomes. Findings from a recent systematic review (Forsman & Nordmyr, 2017) indicated a positive association between internet use and mental health, which was related to enhanced interpersonal interactions, increased community access, and empowered inclusion at the society level. Döring and colleagues (2022) conducted a scoping review of research examining the impact of communication technologies (CTs) on loneliness and social isolation in older adults. Positive impacts of CT use were shown in 55% of the studies assessing loneliness and 44% of those assessing social isolation. Yu et al. (2020) examined the impact of internet use on loneliness among older adults over 8 years and found that internet use was associated with decreased loneliness and more social contact and that social contact was related to lower perceived loneliness. In a longitudinal study, Szabo et al. (2019) examined the indirect impact of online engagement for social, informational, and instrumental purposes on older adults’ well-being via reducing loneliness and supporting social engagement. The findings indicated online social activities indirectly affected well-being via decreased loneliness and increased social engagement. Use of the internet for informational and instrumental purposes indirectly affected well-being through activity engagement, but was unrelated to loneliness. These findings highlight that the impact of technology use may vary depending on the type of use. In this regard, Lam et al. (2020) assessed the impact of the frequency and type of internet use on the emotional well-being of older adults using data from the English Longitudinal Study of Aging. The findings indicated that using the internet for communication was associated with lower depression and better life satisfaction, whereas using the internet for information access was not associated with changes in depression.

Using an iterative, user-centered design approach, researchers developed the Personal Reminder Information and Social Management (PRISM) software application that provided access to resources, information, social engagement, and communication (Czaja et al., 2015). PRISM was evaluated in a multisite randomized controlled trial (RCT) with older adults at risk for social isolation. PRISM participants reported significantly lower levels of loneliness and increased social support and well-being compared to those assigned to a control condition, where participants received the same amount of social contact and a notebook with similar content, at the 6-month follow-up (Czaja et al., 2018). The sample in the PRISM trial was community-dwelling older adults who mostly resided in urban locations. Building on this work, in this study we sought to extend the results of the PRISM trial with a new technology platform (tablet rather than desktop computer) with expanded social features (PRISM 2.0) and examine the impact of access to the system among diverse populations of older adults, in different living contexts. Relative to the previous trial, we set a higher bar for the control condition; people in this condition (Tablet condition) received a computer tablet (without PRISM 2.0) and the same amount of training as those in the PRISM 2.0 condition. The design of the PRISM 2.0 system was based on: theories regarding successful aging (e.g., Liu et al., 2019; Rowe & Kahn, 1998); environmental support (Morrow & Rogers, 2008), the literature regarding age changes in abilities (e.g., procedural and prospective memory loss, Backman et al., 2001); findings from the PRISM trial (Czaja et al., 2018), and from guidance regarding interface design, training, and implementation (e.g., Czaja & Sharit, 2013; Czaja et al., 2019). For example, theories of successful aging indicate the importance of social connectivity and engagement. Features such as Skype and email were included to foster engagement. Findings from the PRISM trial indicated that the participants enjoyed the games features and therefore we included a broarder array of games on PRISM 2.0, including single player and multiplayer games.

Our aims in this study were to examine the impact of access to PRISM 2.0 compared to a standard tablet on psychosocial and technology-related (e.g., technology proficiency) outcomes among older adults living in senior housing, rural areas, and ALCs. We hypothesized that participants receiving PRISM 2.0 would demonstrate greater improvements in social support and connectedness, health-related quality of life, quality of life and reduced loneliness and social isolation than those in the Tablet condition. The trial was conducted to adhere to as far as possible to the Consort Standards for Randomized Clinical Trials. In this article, we focus on the 6-month outcome data, as this timepoint is proximal to when we found the largest impact of the PRISM system (Czaja et al., 2018), and because this timepoint was less affected by disruptions due to the COVID-19 pandemic, which was particularly disruptive for those in the assisted living communities (ALCs).

## Method

### Study Design

The trial was a multisite RCT conducted in Miami, Florida, in ALCs; predominantly rural locations in Leon County, Florida; and senior housing in Atlanta, Georgia. Assessments occurred at baseline and 6 and 9 months postrandomization. The Institutional Review Boards at the three sites approved the study protocol. Participants provided written informed consent and were compensated $30 per assessment. Enrollment occurred on a rolling basis and began in July 2017 and ended in December 2019. Follow-up assessments were disrupted due to mandatory “shut downs” associated with the COVID-19 pandemic, especially in ALCs. Due to the confounds of the COVID-19 pandemic and associated challenges with conducting follow-up assessments, we focus on the 6-month follow-up data.

### Participants

The sample was a convenience sample and included individuals aged 65 and older who spoke English or Spanish; did not have a visual or hearing impairment that could not be corrected (e.g., with glasses or a hearing aid); did not have a motor impairment that would interfere with use of a keyboard; did not have a severe illness that would interfere with participation; were able to read at the 6th grade level (Woodcock-Johnson III Test of Achievement administered at baseline; Wendling et al., 2007); and were at risk for social isolation. During the telephone screening, potential participants were administered the following measures: Telephone Interview for Cognitive Status (TICS; Brandt et al., 1988), and four items from the Mobile Device Proficiency Questionnaire (MDPQ; Roque & Boot, 2018) to assess technology experience. They were also asked four questions about their engagement in work and social activities and about loneliness using three items based from the Hughes et al. (2004) loneliness scale and an additional item developed by the investigators. The cutoff for the TICS was ≤23. For technology experience, participants were excluded if they used a computer, tablet, or email with high frequency and/or if they scored >16 on the combined MDPQ questions. For the social isolation screen, items were scored on a 4-point scale ranging from never to most of the time (score range: 4–16) with a higher score indicating more loneliness. Participants were excluded if they failed to meet two out of the three social isolation criteria (e.g., working >5 hours a week and a score of 3 on the social isolation screen). The cutoff for inclusion was a score ≥6.

In addition, during the baseline assessment participants were administered the Fuld Object Memory Evaluation (FULD; Wall et al., 1998). The cutoff for those aged 65–79 was ≥19 of 30 targets recalled across the three trials and for those ≥80, the cutoff was ≥18 of 19 of 30 targets recalled across the three trials.

Recruitment strategies included: media advertisement, interactions with agencies serving older adults (e.g., Meals on Wheels), use of mailing lists, posting flyers, meeting with staff (senior housing), and working with ALC Directors and Activity Directors.

#### PRISM 2.0 condition

PRISM 2.0 participants received a lightweight LG Tablet (10.1) (~1.07 lbs.) with 10.1-inch 1,920 × 1,200 pixels resolution IPS LCD screen, rear 8 mega-pixel camera, 2 mega-pixel front-facing camera, and a 7400 mAh battery. PRISM 2.0 was an Android application and preloaded onto the tablet, which was connected to a secure server and free internet access was provided through a preloaded Sim wireless card. There were no login requirements to access PRISM 2.0; the application appeared as an icon on the screen.

**Figure 1.**
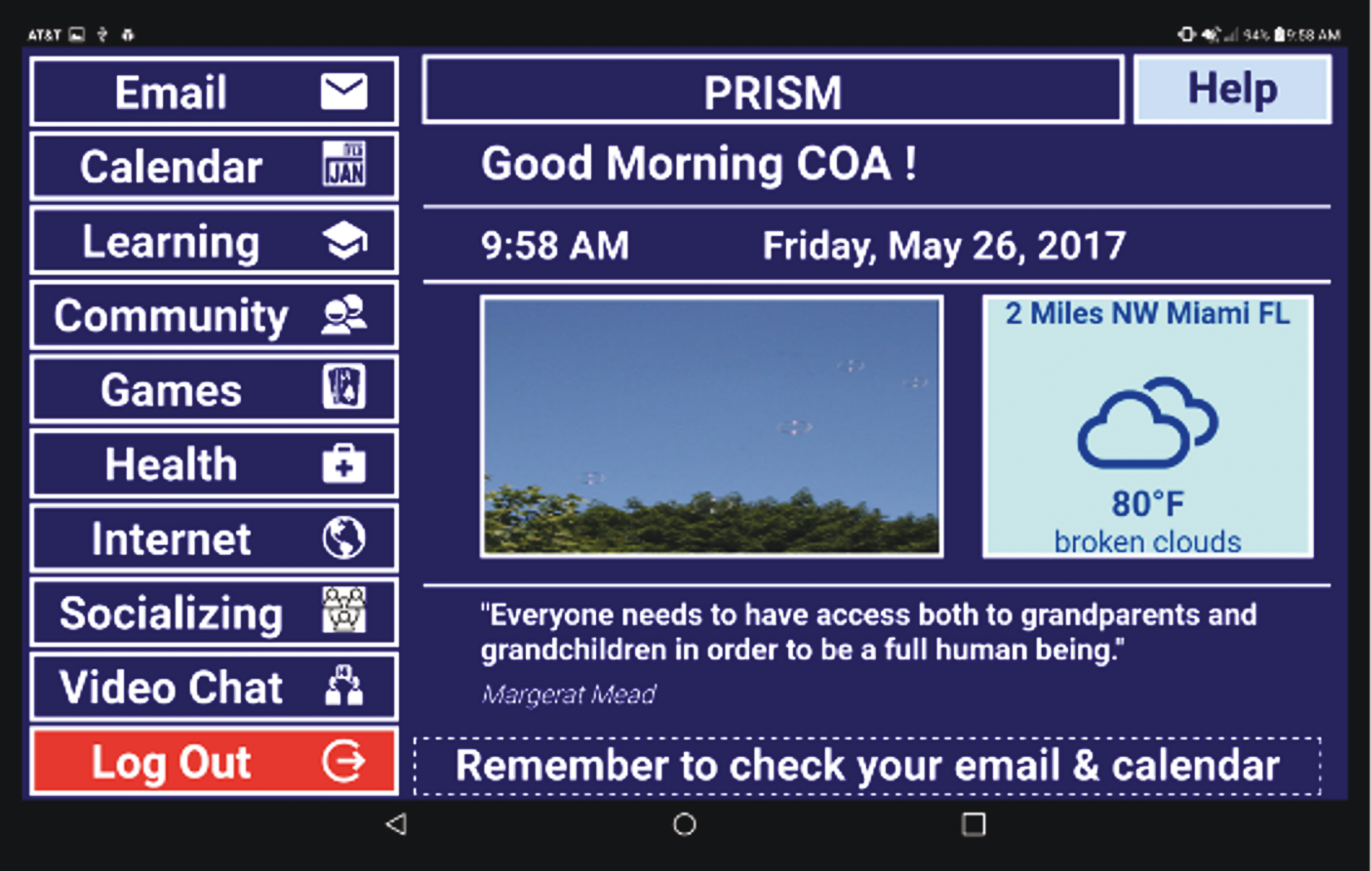
Home page of the PRISM 2.0 system.

The integrated features of PRISM 2.0 included an *email feature* designed to support connectivity and communication; *Internet access* with vetted links to sites such as NIA health topics; a *Classroom feature* intended to augment procedural knowledge and provide new learning opportunities; a *Socializing feature* that provided ways to increase socialization and engage in activities such as volunteering and mentoring; a *Health feature* that included information on health conditions, health-related apps (e.g., BMI calculator), vetted health resources (e.g., WebMd), and a health glossary in simple language; a *Community feature* that included vetted lists of national and local resources and community events; a *Games feature* that included single and multiplayer interactive web-based games; a *Calendar feature* that included a reminder functionality; a *Videochat feature*, Skype; and online help (Figure 1).

PRISM 2.0 was developed using an iterative user-centered design approach that included interviews with prior users of PRISM (frequent and infrequent users) and subject matter experts (e.g., a researcher with expertise in conducting research with aging adults in rural locations, an activities director at a senior housing facility; and a researcher with expertise conducting technology-based research in assisted living facilities); and two rounds of usability testing at each site. The investigators also conducted a heuristic analysis of PRISM 2.0 using current usability guidelines (e.g., Czaja et al., 2019).

### The Tablet Control Condition

Participants in the Tablet Control Condition received the LG tablet without PRISM 2.0 and were provided with SIM-based internet access. They were introduced to information and applications on the tablet similar to those contained in PRISM 2.0, such as SKYPE, Gmail, Calendar, Facebook, Google Play, YouTube, Chrome, and settings via native Android applications. Participants received that same planned contact as those in the PRISM 2.0 condition, including the same amount of individual training and technical support.

### Protocol

Interested participants completed a telephone eligibility screening and those eligible and interested were scheduled for an in-person baseline assessment (prepandemic). Those that remained eligible at baseline were assigned to a study condition. Randomization was done at the individual level at the Leon County site and at the facility level at the Miami and Atlanta sites to avoid within-facility contamination. At the Miami site, 11 ALCs agreed to participate, located across the greater Miami area that varied in race/ethnicity (some were primarily Hispanic), size, and income strata. At the Atlanta site, 11 geographically dispersed senior housing facilities agreed to participate. Staff were supportive of the randomization as all participants received technology and training as well as technical support by the study investigators.

Participants in both conditions received three individual interactive training sessions, were given practice homework, received check-in calls 1 week and 1 month following training, were provided with a user manual and a help card, and had access to technical help. They also received check-in calls at 3 and 7 months. If there were 3 weeks of continuous nonuse of PRISM 2.0 or the standard Tablet, participants were contacted to determine if additional support was needed.

Participants completed follow-up assessment at 6 and 9 months postrandomization. They could keep the tablet postintervention; however, PRISM 2.0 and free internet service were no longer available. They were offered help to procure and install a commercial internet service if desired.

An assessor blinded to treatment condition administered the primary and secondary outcome measures in-person at 6 and 9 months in-person (prepandemic) or over the telephone during the COVID-19 pandemic. The same assessor mailed the instruments that were self-administered (e.g., mobile device proficiency questionnaire).

### Treatment Fidelity

All sites used a detailed manual of operation procedures and applied equivalent procedures and standardized protocols. We also developed manuals for the administration of the assessment battery. In addition, the research assistants were provided with scripts for each participant’s interaction (e.g., screening, assessments, training, check-in calls, and so on). There were cross-site monthly project coordinator and bi-weekly investigator conference calls. Assessor and interventionist training included training on the administration of the assessment battery and protocols for both study conditions. This training encompassed role-playing on both the administration of the assessment battery and training of the participants. These procedures were adapted from the highly successful PRISM 1.0 trial.

### Measures

#### Primary and secondary outcome measures

The primary outcome measures included changes, at 6 months, in: loneliness (UCLA Loneliness Scale; Russell, 1996); perceived social support (MOS Social Support Survey; Ware et al., 1992); social connectedness (Social Connectedness Scale; Lee & Robbins, 1995); perceptions of quality of life (WHO Quality of Life Scale; World Health Organization, 1995); and health-related quality of life (The SF-36 Health Survey [modified]; Ware & Sherbourne, 1992).

Secondary outcome measures included changes in social isolation (perceived social isolation; Cornwall & Waite, 2009), technology readiness (Technology Readiness Index; Parasuraman & Colby, 2015), and mobile device proficiency, (Mobile Device Proficiency Scale; Roque & Boot, 2018).

PRISM usage data included the number of times participants accessed one of the eight feature categories of PRISM in a day. Total usage was the sum of the daily usage scores across days. Time spent using the system and features was recorded but not included in the analyses as a feature may have been accessed but the participant may have engaged in other activities while leaving the feature active. Thus, time could reflect an inflated indicator of use.

### Analyses

The primary outcomes were analyzed using multilevel mixed models testing the effect of condition, time, and their interaction while treating the intercept as a random effect. There were only two time points analyzed (baseline and 6-month follow-up); thus, time was treated as a fixed effect. Each site represented a different type of living context: rural community living (Tallahassee); senior housing (Atlanta); and ALCs (Miami), and there were systematic differences among the sites (Tables 1 and 2) on several variables; therefore, we analyzed the primary and secondary outcomes for each site separately. Supplementary Table 1 presents the beta weights and *p* values for the outcome variables by living location.

**Table 1.** Participant Demographics by Living Situation and Study Condition^a^

Variables	Living situation				Study condition		
	Senior housing	Rural locations	ALCs	Significance	PRISM 2.0 (*n*)	Tablet (*n*)	Significance
	n	n	n		n	n	
Race/ethnicity				χ^2^(6) = 62.81, *p* < .001			χ^2^(3) = 19.70, *p* < .001
Hispanic	5	0	21		23	3	
White Caucasian	46	65	54		71	94	
Black/African American	29	16	0		26	19	
Other	5	3	1		5	4	
Gender				χ^2^(6) = 4.55, *p* = .10			χ^2^(1) = 1.88, *p* = .17
Male	17	14	23		23	31	
Female	67	70	53		101	89	
Education				χ^2^(16) = 21.16, *p* = .17			χ^2^(8) = 4.36, *p* = .82
Less than high school	6	1	2		4	5	
High school graduate/GED	10	10	7		12	15	
Vocational training/some college	40	27	31		48	50	
Bachelor’s degree or higher	27	46	36		59	50	
Income				χ^2^(6) = 41.7, *p* < .001			χ^2^(3) = 1.6, *p* = .66
Less than $20,000	58	23	17		48	50	
$20,000–$39,999	14	22	14		28	22	
$40,000–$69,999	2	20	16		21	17	
>$70,000	2	7	5		9	5	
Marital status				χ^2^(8) = 61.34, *p* < .001			χ^2^(3) = 2.65, *p* = .62
Single	22	12	7		20	21	
Married	0	2	9		5	6	
Separated/divorced	41	38	9		44	44	
Widowed	20	29	51		54	46	
Health				χ^2^(8) = 12.02, *p* = .15			χ^2^(3) = 1.62, *p* = .81
Excellent/very good	30	41	28		50	49	
Good	34	28	32		48	46	
Fair/poor	19	7	12		18	20	

*Notes*: ALC = assisted living community; GED = General educational diploma.

^a^The *n*’s may be unequal due to missing data.

**Table 2.** Descriptive Statistics: Age, MOCA, and Outcome Measures by Living Situation and Condition

Variables	Living situation				Study condition		
	Senior housing	Rural locations	ALCs	*F* value	PRISM 2.0	Tablet	*F* value
	*m* (*SD*)	*m* (*SD*)	*m* (*SD*)		*m* (*SD*)	*m* (*SD*)	
Age	74.42 (7.03)	73.70 (5.86)	85.77 (7.76)	***F*(2,243) = 72.52, *p* < .001**	77.78 (8.58)	77.60 (9.03)	*t*(243) = −0.17, *p* = .4
MOCA	23.66 (3.97)	25.36 (3.01)	23.61 (2.93)	***F*(2,235) = 7.01, *p* < .001**	24.46 (3.33)	24.00 (3.56)	*t*(236) = 1.03, *p* = .15
Loneliness	39.89 (9.60)	40.79 (9.97)	40.05 (9.47)	*F*(2,235) = .18, *p* = .84	39.84 (9.28)	40.67 (10.06)	*t*(236) = 0.67, *p* = .25
Quality of life	23.27 (3.54)	23.16 (3.71)	22.65 (3.71)	*F*(2,236) = .53, *p* = .59	23.24 (3.71)	22.83 (3.59)	*t*(238) = −0.89, *p* = .19
Health-related quality of life	856.96 (168.48)	881.42 (189.65)	817.35 (181.71)	*F*(2,229) = 1.39, *p* = .25	846.18 (194.10)	860.19 (168.05)	*t*(230) = 0.27, *p* = .39
Social support	61.50 (19.42)	60.41 (21.82)	63.63 (21.10)	*F*(2,237) = .46, *p* = .63	60.98 (20.96)	62.59 (20.58)	*t*(238) = 0.61, *p* = .27
Social isolation	14.20 (2.49)	14.27 (2.41)	14.91 (2.44)	*F*(2,236) = 2.24, *p* = .11	14.35 (2.38)	14.54 (2.55)	*t*(237) = 0.50, *p* = .31
Technology readiness	49.57 (8.02)	45.79 (7.82)	50.00 (8.25)	***F*(2,219) = 6.74, *p* < .001**	48.90 (8.40)	47.91 (7.93)	*t*(220) = −0.83, *p* = .21
Mobile device proficiency	21.72 (8.78)	21.62 (8.85)	16.87 (8.70)	***F*(2,218) = 7.85, *p* < .001**	20.35 (8.91)	19.97 (9.16)	*t*(219) = −0.27, *p* = .39

*Note*: ALC = assisted living community; MOCA = Montreal Cognitive Assessment. Bolded results are statistically significant.

We used the same multilevel mixed modeling approach to examine the effect of system usage on outcomes for those participants within the PRISM 2.0 condition. We did not have usage data for those in the Tablet condition due to constraints associated with the internet provider. We entered three variables into the model: usage, time, and the usage by time interaction. We tested the effect of total usage, time (baseline and 6 months), and the interaction of usage with time. As expected, the total usage variable was highly skewed and so for analysis purposes a log10 transformation was applied.

We used path analysis, using change scores, to assess the causal pathways between changes in our social variables (social isolation and support), loneliness, quality of life, and health-related quality of life. Based on findings from the PRISM trial (Czaja et al., 2021), our hypothesized model predicted that changes in social isolation and support would lead to changes in loneliness, which in turn would be related to changes in quality of life and health-related quality of life. We also hypothesized that the relationship of the social variables to the outcome variables would be mediated by loneliness. We created a latent variable for social support from the tangible, affective, and informational subscales of the MOS. The connectiveness subscale did not load significantly onto the latent variable and thus was not included. We used bootstrap confidence intervals to estimate the indirect effect and for simplicity used those confidence intervals for parameter inferences, where betas are the standardized parameter.

As is common in all complex community-based interventions, some missing data were expected. At baseline, missing data ranged from 1% to 3% for the psychosocial outcomes. Some participants did not return completed background information forms, administered via mail, and data related to technology experience were missing for 10% of the participants.

To characterize the sample, we included all eligible randomized participants providing baseline data. For analyses of intervention effects, we included participants with both baseline and 6-month data. If participants had missing data on some measures at 6 months, values were imputed. We chose to use multiple imputation (SPSS 26) because as noted by Li et al. (2015), multiple imputation provides accurate estimates of quantities or associations of interest, such as treatment effects in randomized trials, and reduces the chance of false-positive or false-negative conclusions. We imputed five data sets. SPSS uses a Sauterwaite approximation to calculate degrees of freedom for mixed model analyses, and we report the degrees of freedom from the 5th imputation.

When imputing data, values outside of the range are possible, thus we *z*-scored each item before forming scale composites. *Z*-scoring the items also simplifies the computations in the multilevel analysis.

### Sampl

**Figure 2.**
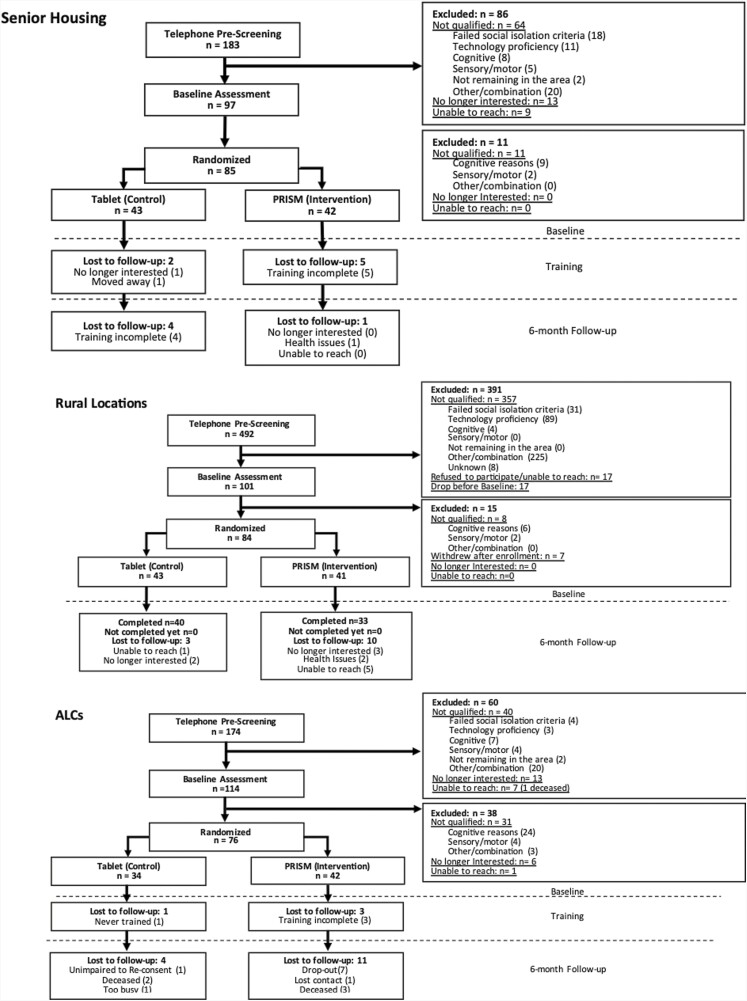
Site consort diagrams. ALC = assisted living community.

Figure 2 presents the site consort diagrams. As shown, 34% of participants in ALCs, 47% in senior housing, and 79% in rural communities were excluded during the telephone screening. A significantly greater number of participants in ALCs (33%) were excluded at baseline as compared to those in senior housing (11%) and rural communities (15%) (χ^2^(2) = 18.15, *p* < .001) and a significantly higher number of people within the ALCs (21%) were excluded due to cognitive reasons as compared to those within senior housing (9%) and within rural locations (6%), (χ^2^(2) = 13.44, *p* = .001). As expected, those in ALCs were frailer than the other participants.

Two hundred and forty-five participants were randomized including 76 participants in ALCs, 85 participants in senior housing, and 84 participants in rural locations. Across the sites, the sample was primarily female (78%), fairly well educated (85%) had beyond a high school education, and 62% reported good to excellent health. Five percent reported that English was not their primary language. All training and assessment materials, and content of the PRISM 2.0 and the Tablet were available in English and Spanish.

Participants within the ALCs were older, Hispanic, widowed and had significantly lower mobile device proficiency as compared to those in the other living settings. On average, participants in senior housing had substantially lower incomes. Participants within rural locations reported less technology readiness; however, these participants had higher Montreal Cognitive Assessment scores (Tables 1 and 2).

The PRISM condition had a greater proportion of Hispanic participants, χ^2^(3) = 19.72, *p* < .001. In Miami, randomization was by ALC, and the ALC with the largest number of Hispanic participants was randomized to the PRISM condition (Tables 1 and 2).

### Attrition at 6 Months

Attrition at the 6-month follow-up was 25%, 23%, and 14% within the ALCs, rural locations, and senior housing, respectively. Within the ALCs, 63% of those were lost to follow-up due to health issues or death, which was significantly higher (χ^2^(2) = 7.80, *p* = .02) than that within rural locations (11%) or senior housing (33%). Across the sample, the results indicated that being older was significantly associated with attrition (OR = 1.04, *p* = .04) and as noted ALC participants were also significantly older (*F*(2,243) = 72.52, *p* < .001).

## Results

### Primary Outcomes

#### Loneliness

There was a significant decrease in loneliness among participants in both the PRISM 2.0 and Tablet conditions in senior housing (*b* = −0.69, 95% CI [−1.20, −0.18], *t*(76) = −2.66, *p* = .008) and rural locations (*b* = −1.02, 95% CI [−1.48, −0.56], *t*(75) = −4.38, *p* < .001) over time. However, over time, there was no change in loneliness (*b* = −0.23, 95% CI [−0.79, 0.33], *t*(64) = −0.79, *p* = .43) for those in ALCs. There was no effect of condition for those in senior housing (*b* = −3.42, 95% CI [−8.55, 1.71], *t*(121) = 1.31, *p* = .19), rural locations (*b* = −1.08, 95% CI [−6.36, 4.19], *t*(109) = 0.40, *p* = .69), or in ALCs (*b* = −1.68, 95% CI [−3.78, 7.14], *t*(103) = 0.60, *p* = .55). Nor was there a significant condition by time interaction for those in senior housing (*b* = 0.44, 95% CI [−0.58, 1.46], *t*(76) = 0.85, *p* = .40); rural locations (*b* = 0.29, 95% CI [−0.64, 1.21], *t*(75) = 0.61, *p* = .54); or ALCs (*b* = −0.05, 95% CI [−1.16, 1.06], *t*(64) = −0.08, *p* = .93). Overall, the data indicate that both technology platforms resulted in improvement in loneliness, but only among those living in rural locations and senior housing.

#### Quality of life

Across both conditions, participants in senior housing (*b* = 0.3, 95% CI [0.1, −0.5], *t*(73) = 3.1, *p* = .002) and in rural locations (*b* = 0.3, 95% CI [0.07, 0.5], *t*(76) = 2.7, *p* = .007) reported a significant improvement in quality of life over time. For those in ALCs, there was no change in quality of life over time (*b* = 0.006, 95% CI [−0.2, 0.2], *t*(59) = −0.05, *p* = .96). There was no effect of condition (*b* = 0.9, 95% CI [−1.0, 2.8], *t*(118) = 1.0, *p* = .3) for those in senior housing, in rural locations (*b* = −0.2, 95% CI [−0.6, 0.2], *t*(120) = 1.7, *p* = .1), or in ALCs (*b* = −1.1, 95% CI [−3.2, 1.0], *t*(101) = −1.0, *p* = .3). The time by condition interaction was not significant for those in senior housing (*b* = −0.2, 95% CI [−0.6, 0.2], *t*(73) = −0.9, *p* = .4), rural locations (*b* = −0.2, 95% CI [−0.6, 0.2], *t*(76) = −1.0, *p* = .3) or those in ALCs (*b* = −0.1, 95% CI [−0.6, 0.3], *t*(50) = −0.6, *p* = .5). Results mirror those of loneliness, with both technology platforms associated with improvements in quality of life over time, but only among those in rural and senior housing locations.

#### Social support

Over time and across both conditions, participants in senior housing (*b* = 0.20, 95% CI [0.04, 0.36], *t*(75) = 2.47, *p* = .01) and in rural locations (*b* = 0.21, 95% CI [0.03, 0.38], *t*(78) = 2.30, *p* = .02) reported significant increases in social support. Among ALC participants, there was no change in social support over time (*b* = 0.03, 95% CI [−0.19, 0.24], *t*(65) = 0.24, *p* = .81). The effect of condition was not significant for those in senior housing (*b* = 0.52, 95% CI [−1.00, 2.05], *t*(122) = 0.67, *p* = .50), or rural locations (*b* = 0.13, 95% CI [−1.5, 1.8], *t*(128) = 0.16, *p* = .87). However, ALC participants in the tablet condition reported higher levels of social support (*b* = −1.94, 95% CI [−3.71, −0.17], *t*(116) = −2.15, *p* = .03). The interaction of time and condition was not significant for those in senior housing (*b* = 0.09, 95% CI [−0.24, 0.41], *t*(75) = 0.53, *p* = .60), rural locations (*b* = −0.09, 95% CI [−0.45, 0.26], *t*(77) = −0.50, *p* = .62) or among those in ALCs (*b* = 0.13, 95% CI [−0.30, 0.57], *t*(65) = 0.60, *p* = .55.

#### Health-related quality of life

Participants in senior housing across both conditions reported significant improvements in health-related quality of life over time (*b* = 0.02, 95% CI [0.004, 0.04], *t*(76) = 2.35, *p* = .02). For participants in rural locations (*b* = −0.003, 95% CI [−0.02, 0.02], *t*(77) = −.34, *p* = .73), or in ALCs (*b* = −0.02, 95% CI [−0.04, 0.004], *t*(65) = −1.64, *p* = .10) there was no change in health-related quality of life over time. Further, there was no effect of condition for those in senior housing (*b* = 0.01, 95% CI [−0.19, 0.21], *t*(117) = 0.07, *p* = .94), in rural locations (*b* = 0.13, 95% CI [−0.06, 0.32], *t*(125) = 1.36, *p* = .18), or in ALCs (*b* = −0.20, 95% CI [−0.42, 0.02], *t*(105) = 1.74 *p* = .08). The interaction of time and condition was not significant for those in senior housing (*b* = 0.02, 95% CI [−0.02, 0.05], *t*(76) = 0.79, *p* = .43), rural locations (*b* = −0.02, 95% CI [−0.06, 0.02], *t*(76) = −1.05, *p* = .29) or in ALCs (*b* = 0.04, 95% CI [−0.006, 0.09], *t*(64) = −1.71, *p* = .09).

### Secondary Outcomes

#### Social isolation

There were significant reductions in social isolation among participants in senior housing (*b* = 0.24, 95% CI [0.09, 0.4], *t*(75) = 3.21, *p* = .001) and in rural locations (*b* = 0.27, 95% CI [0.12, 0.43], *t*(78) = 3.42, *p* = .001) in both conditions over time but not for those in ALCs (*b* = −0.02, 95% CI [−0.18, 0.15], *t*(65) = −0.18, *p* = .85). There was no effect of condition for those in senior housing (*b* = 0.006, 95% CI [−1.29, 1.30], *t*(129) = .009, *p* = .99), rural locations (*b* = −0.83, 95% CI [2.17, 0.52], *t*(131) = −1.20, *p* = .23), or ALCs (*b* = −0.11, 95% CI [−1.57, 1.34], *t*(65) = −0.16, *p* = .88). The interaction between time and condition was not significant for those in senior housing (*b* = −0.05, 95% CI [−0.23, 0.34], *t*(75) = − 0.37, *p* = .71), rural locations (*b* = −0.23, 95% CI [−0.08, 0.54], *t*(78) = −1.46, *p* = .15) or ALCs (*b* = −0.13, 95% CI [−0.46, 0.20], *t*(65) = −0.79, *p* = .43).

#### Mobile device proficiency

Mobile device proficiency significantly improved among participants in senior housing (*b* = 1.66, 95% CI [1.21, 2.11], *t*(75) = 7.28, *p* < .001), rural locations (*b* = 1.97, 95% CI [1.59, 2.36], *t*(74) = 10.07, *p* < .001), and in ALCs (*b* = 1.59, 95% CI [1.08, 2.04], *t*(63) = 6.3, *p* < .001) across both conditions over time. The effect of condition was not significant for those in senior housing (*b* = 2.60, 95% CI [−2.12, 7.32], *t*(115) = 1.08, *p* = .28), rural locations (*b* = −3.61, 95% CI [−0.8.31, 1.33], *t*(106) = −1.51, *p* = .13), or ALCs (*b* = 3.92, 95% CI [−1.05, 8.89], *t*(100) = 1.55, *p* = .12). Finally, the interaction of time and condition was not significant for those in senior housing (*b* = −0.83, 95% CI [−1.74, 0.08], *t*(75) = −1.79, *p* = .08), rural locations (*b* = 0.55, 95% CI [−0.23, 1.33], *t*(74) = 1.39, *p* = .17), or ALCs (*b* = −.11, 95% CI [−1.09, 0.85], *t*(63) = −.23, *p* = .82).

#### Technology readiness

Among participants in senior living (*b* = −0.05, 95% CI [−0.45, 0.34], *t*(79) = −0.25, *p* = .80), or ALCs (*b* = −0.35, 95% CI [−0.71, 0.01], *t*(62) = −1.90, *p* = .06) there was no significant change in technology readiness over time. In contrast, there was a significant increase in technology readiness among those in rural locations over time (*b* = 0.43, 95% CI [0.10, 0.76], *t*(71) = 2.53, *p* = .01). There was no condition effect for technology readiness for those in senior housing (*b* = 2.76, 95% CI [−0.76, 6.29], *t*(132) = 1.54, *p* = .12), rural locations (*b* = −0.58, 95% CI [−4.09, −2.92], *t*(114) = −0.32, *p* = .74), or ALCs (*b* = 0.17, 95% CI [−3.55, 3.89], *t*(97) = 0.09, *p* = .93). Further, the time and condition were not significant for those in senior housing, rural locations (*b* = −0.31, 95% CI [−1.00, 0.39], *t*(71) = −0.87, *p* = .39), or in ALCs (*b* = 0.3, 95% CI [−0.40, 0.99], *t*(62) = 0.83, *p* = 0.41).

### Usage of PRISM 2.0

As an exploratory analysis, we examined the association of use of the PRISM 2.0 system across all PRISM participants and our outcomes. Supplementary Table 2 reports the unstandardized betas and *p* values for the time by usage interactions. Across PRISM participants, quality of life increased significantly more over time with greater use of the system (*b* = 0.42, 95% CI [0.007, 0.83], *t*(95) = 2.00, *p* = .046) as did mobile device proficiency based on a one-tail test (*b* = 0.81, 95% CI [−0.07, 1.68], *t*(95) = 1.82, *p* = .07).

### Path Analyses

Decreases in social isolation were associated with increases in social support (β = −0.29, 95% CI [−0.46, −0.12]) and as hypothesized, decreases in both social isolation (β = 0.38, 95% CI [0.24, 0.52]) and increases in social support (β = −0.25, 95% CI [−0.43, −0.07]) were significantly associated with decreases in loneliness.

Overall, both changes in social isolation (β = −0.33, 95% CI [−0.49, −0.16]) and loneliness (β = −0.25, 95% CI [−0.40, −0.11]) had a direct effect on changes in quality of life at 6 months and loneliness mediated the relationship between social isolation and loneliness (β = −0.10, 95% CI [−0.17, −0.03]). The indirect effect of social isolation on changes in quality of life through social support and then through loneliness was not significant (β = −0.02, 95% CI [−0.04, 0.003]). Social support did not have a direct effect (β = 0.15, 95% CI [−0.03, 0.34]) on changes in quality of life but did have an indirect effect on changes in quality of life through loneliness (β = 0.06, 95% CI [0.03, 0.13]).

**Figure 3.**
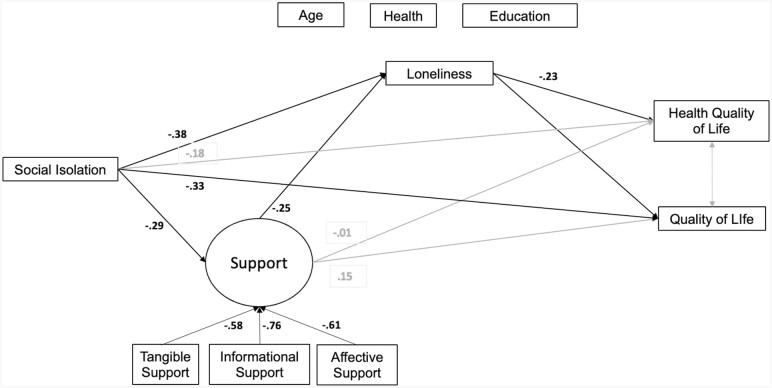
Mediation model controlling for age, self-reported health, and education level. Bold lines and numbers represent significant paths. Light gray lines and numbers represent not-significant paths. All exogenous and endogenous indicators represent the change score calculated as *t*2−*t*1 for each variable.

Loneliness (β = −0.23, 95% CI [−0.38, −0.08]) had a direct effect on health-related quality and mediated the relationship between social isolation and health-related quality of life (β = −0.01, 95% CI [−0.02, −0.003]). Changes in social isolation (β = −0.18, 95% CI [−0.39, 0.04]) or in social support (β = 0.01, 95% CI [−0.19, 0.21]) did not directly impact health-related quality of life. The path of social isolation through social support and then loneliness was not significant (β = −0.02, 95% CI [−0.04, 0.01]). Loneliness mediated the relationship between social support and health-related quality of life (β = 0.04, 95% CI [0.001, 0.12]). Figure 3 presents the model parameter estimates. The model fit was excellent (χ^2^(17) = 20.4, *p* = .3, CFI = 0.99).

## Discussion

This study examined the impact of a software system, PRISM 2.0, on psychosocial and technology-related outcomes among a sample of older adults in diverse living settings. For participants in senior housing and rural locations use of both PRISM 2.0 and a computer tablet resulted in decreases in loneliness and social isolation and increases in quality of life and perceived social support over time. Participants in senior housing reported an increase in health-related quality of life. These findings are consistent with those of the PRISM trial (Czaja et al., 2018) and of others (Cotten et al., 2014; Forsman & Nordmyr, 2017; Szabo et al., 2019; Yu et al., 2020), indicating that technology can play an important role in enhancing opportunities for social engagement and decreasing loneliness and isolation among older adults. It is well documented that social isolation and loneliness are common issues among older adults, especially since the COVID pandemic, and can have detrimental consequences on cognitive, physical, and emotional health (National Academies of Science Engineering and Medicine, 2020).

In contrast to our hypothesis, there was no added beneficial effect of the PRISM 2.0 system as compared to a standard tablet. However, as noted, participants in the tablet condition received 3 days of individual training; had well-designed support materials and easy access to technical help; and received periodic check-in calls. Further, the training protocol was pilot tested prior to implementation. The tablet training protocol we employed is not typical as most people encounter new technology with minimal amounts of training and often instructions regarding use are online and challenging to access. Findings from the Pew Research Center (Smith, 2014) indicated that older adult non-internet users lack confidence in their ability to use the internet and expressed a need for support. Theoretical models such as the Unified Theory of Acceptance and Use of Technology (Venkatesh et al., 2012) that identify social support as a predictor of technology acceptance and use, and previous findings (e.g., Haase et al., 2021; Sharit et al., 2021) indicate that among older adults, available support from family and friends is associated with willingness to adopt new technology. Providing older adults with appropriate training and technical support when introducing new technology, especially for those with limited technology skills such as our participants, is critically important.

PRISM 2.0 was designed with a user-centered design approach, yet there were technical challenges with the system during the trial. We relied on Google servers for our calendar and email apps and OAuth procedures for signing into those servers. Unfortunately, protocols changed at Google, and we had to retrain a subset of the sample to use the Google calendar and Gmail web-based apps rather than our apps. This change disrupted use of email for a time period, which likely affected system use. Microsoft also changed the Skype interface and thus we needed to modify our use instructions and technical support protocols. However, despite these challenges, across our participants, greater use of the PRISM 2.0 system resulted in greater improvements in quality of life and mobile device proficiency over time.

Although we did not have a hypothesis in regards to living context, the study was designed to explore potential differences in benefits of technology in diverse living contexts. Use of either PRISM 2.0 or the standard Tablet did not have an impact on the psychosocial outcomes among the ALC participants. These participants were significantly older, and a greater proportion were lost to follow-up due to illness or death. Further, a greater number of participants were excluded from the trial due to cognitive challenges. Levine et al. (2018) found that use of everyday and digital health technologies lessened among older adults with declining health. Seifert and colleagues (2017) found that adults aged 65–84 years in residential facilities (RCF) had lower internet use compared with community-dwelling older adults, and that compared with nonusers, internet users in RCFs were more likely to be younger, male, living for a shorter duration in RCF, not living alone, healthier, and functionally unimpaired. Our sample was primarily female, older, and less healthy. Our findings are somewhat in contrast to those of Cotten et al. (2013), who found that going online decreased loneliness among those in assisted living facilities but had no impact on social isolation. This may be due to the differences in the training protocol. In the Cotten et al. (2013) study, participants were trained in groups over 8 weeks, and the focus was on using the internet to communicate and find information. Our training was shorter and focused on a greater number of features.

In addition to person factors that may have limited the benefits of technology access for those in ALCs, there were challenges at the facilities such as staff turnover; maintaining staff engagement, especially during COVID; and challenges scheduling training and assessments due to facility constraints. Training generally took longer with these participants due to fatigue. For this population, more training and shorter training sessions spaced over time would likely be beneficial, as would frequent rest breaks.

Among all participants, including those in the ALCs, there was a significant improvement in mobile device proficiency over time. Even among the older cohorts, new technologies can be learned. Across the sites, 23% of the sample was 85 and older and of these, 47% were greater than 90 years. We also found in the PRISM trial (Czaja et al., 2018) that technology skills improved with use of the PRISM software. The findings also support that there is cognitive plasticity in older age.

Our findings underscore the importance of loneliness to quality of life and well-being. Decreased loneliness was directly associated with improvements in quality of life and health-related quality of life. Similar to Szabo and colleagues (2019), we found that the impact of reduced social isolation on changes in quality of life was mediated by decreases in loneliness. Understanding mechanisms of action within an intervention trial is critical to the refinement and development of future interventions. Our findings suggest that interventions that target loneliness are warranted with respect to improving the quality of life and well-being of older adults. As noted by the Surgeon General of the United States (Murthy, 2023), loneliness is a serious public health problem and there is an urgent need to develop strategies to mitigate loneliness and isolation.

Limitations of the study include that the sample was a convenience sample and that the living contexts were only in one geographical location. Thus, the generalizability of the findings is limited. However, the results do replicate those of other investigators who have found that access to technology is beneficial to aging adults. Further, PRISM 2.0 had technical challenges due to changes within our outsourced providers. Also, like many, we were faced with challenges associated with the COVID-19 pandemic. A significant challenge was the lockdown, which was especially significant at the ALCs. This precluded in-person assessments; all assessments had to be shifted to a mail/telephone protocol. Even with remote administration of the measures, the lockdown also affected administration support from the ALC staff with respect to facilitating interactions with participants (e.g., delivering the mail packet of measures, scheduling the telephone assessments). Overall, the COVID-19 pandemic especially affected the 9-month follow-up data.

Our findings suggest that older adults in ALCs may need a simpler system than PRISM 2.0 and more training, technical support, and planned opportunities for interaction (e.g., group training). Regardless of these challenges, the results demonstrated that older adults, including those in the older cohorts, are willing to use, can learn, and benefit from technology.

## Supplementary Material

igae042_suppl_Supplementary_Tables

## Data Availability

Data will be made available for secondary analysis. Interested parties should contact the corresponding author, Sara J. Czaja.
