# Supplementary material for: The Benefits of Technology for Engaging Aging Adults: Findings From the PRISM 2.0 Trial
Source: Innov Aging. 2024 Apr 25;8(6):igae042. doi: 10.1093/geroni/igae042 (PMC11154146; doi:10.1093/geroni/igae042)
Supplement: igae042_suppl_Supplementary_Tables [file igae042_suppl_supplementary_tables.docx]

***Innovation in Aging* Supplementary Material. Sara J. Czaja, Neil Charness, Wendy A. Rogers, Joseph Sharit, Jerad Moxley, & Walter R. Boot. The Benefits of Technology for Engaging Aging Adults: Findings from the PRISM 2.0 Trial.**

**Supplementary Tables**

**Supplementary Table 1:** Effect of study across time (both conditions) by Living Situation.

| **Variables** | **Senior Housing** | | **Rural Locations** | | **ALCs** | |
| --- | --- | --- | --- | --- | --- | --- |
|  | B (time) | p value | B (time) | p value | B (time) | p value |
| Sf36 | **0.02** | **0.02** | -0.003 | 0.7 | -0.02 | 0.1 |
| Loneliness | **-0.7** | **0.008** | **-1.0** | **<.001** | -0.2 | 0.4 |
| Quality of Life | **0.3** | **0.002** | **0.3** | **0.007** | 0.01 | 0.96 |
| Perceived Isolation | **0.2** | **0.001** | **0.3** | **<.001** | -0.02 | 0.9 |
| MOS-Social Support | **0.2** | **0.02** | **0.1** | **0.02** | 0.03 | 0.8 |
| CESD | -0.3 | 0.2 | 0.2 | 0.3 | 0.5 | 0.09 |
| Technology Readiness | -0.05 | 0.8 | **0.4** | **0.01** | -0.3 | 0.06 |
| Mobile Device Proficiency | **1.6** | **<.001** | **2** | **<.001** | **1.6** | **<0.001** |

**Supplementary Table 2:** Interaction of Total Systems

Uses and Time in the Prism Condition

| **Variables** | B (TimeXUse) | p value |
| --- | --- | --- |
| Sf36 | 0.00008 | 0.2 |
| Loneliness | -0.0003 | 0.9 |
| Quality of Life | **0.001** | **0.04** |
| Percieved Isolation | 0.0002 | 0.7 |
| MOS-Social Support | -0.0002 | 0.7 |
| CESD | -0.002 | 0.1 |
| Technology Readiness | 0.001 | 0.2 |
| Mobile Device Proficency | **0.03** | **0.03** |
